# Clinical Effectiveness of Targeted Therapies Following Nivolumab Therapy in Patients with Metastatic Renal Cell Carcinoma: A Real-World Study

**DOI:** 10.3390/medicina60071088

**Published:** 2024-07-02

**Authors:** Deniz Işık, Oğuzcan Kınıkoğlu, Goncagül Akdağ, Yunus Emre Altıntaş, Ezgi Türkoğlu, Sedat Yildirim, Heves Sürmeli, Tuğba Başoğlu, Hatice Odabaş, Nedim Turan

**Affiliations:** Department of Medical Oncology, Health Science University, Kartal Dr. Lütfi Kirdar City Hospital, Istanbul 34865, Turkey; ogokinikoglu@yahoo.com (O.K.); akdaggoncagul@gmail.com (G.A.); yunusaltintas1688@gmail.com (Y.E.A.); ezgiturk_90@hotmail.com (E.T.); rezansedat@hotmail.com (S.Y.); hevessurmeli@hotmail.com (H.S.); basoglutugba@gmail.com (T.B.); odabashatice@yahoo.com (H.O.); turan.nedim@hotmail.com (N.T.)

**Keywords:** renal cell carcinoma, nivolumab, targeted therapy, efficacy and toxicity

## Abstract

*Background*: The treatment and escape for metastatic renal cell carcinoma (RCC) has rapidly evolved, particularly with the integration of immune therapies into first-line regimens. However, optimal strategies following progression in first-line immunotherapy remain uncertain. This study aims to evaluate the efficacy and safety of axitinib and cabozantinib as third-line therapies after progression on nivolumab following first-line VEGF-TKI therapy. *Methods*: Patients with metastatic RCC who progressed on prior nivolumab treatment after receiving first-line VEGF-TKI therapy were included. Data on patient characteristics, treatment regimens, response rates, progression-free survival (PFS), and overall survival (OS) were collected. Statistical analyses were conducted to assess the prognostic factors and treatment outcomes. *Results*: A total of 46 patients were included who were predominantly male (83%) with clear-cell histology (89%). The median PFS on first-line TKI therapy was 10.2 months. All the patients received nivolumab as a second-line therapy, with a median of 12 cycles. The median second-line PFS was seven months. Third-line therapies included axitinib (24 patients) and cabozantinib (20 patients). The median PFS for axitinib and cabozantinib was six months, with comparable survival outcomes. The IMDC risk group and treatment tolerability were significant predictors of survival in multivariate analysis. Adverse events were manageable, with hypertension, fatigue, and diarrhea being the most common. *Conclusion*: Axitinib and cabozantinib show promise as third-line therapies post-nivolumab progression in metastatic RCC, though prospective validation is warranted. This study underscores the need for further research to establish treatment standards in this evolving landscape.

## 1. Introduction

The treatment of metastatic renal cell carcinoma (RCC) has evolved rapidly since the approval of the first targeted therapy in 2006. In the past two decades, vascular endothelial growth factor tyrosine kinase inhibitors (VEGF-TKIs) have been the mainstay of treatment, demonstrating efficacy in numerous trials, both as single agents and in combination [1]. Currently, immune checkpoint inhibitor (ICI) combinations are the standard first-line therapy in many countries. The Keynote-426 trial demonstrated a significant survival advantage for the pembrolizumab + axitinib combination compared to sunitinib in patients with intermediate and poor risk factors [2]. The Checkmate-214 trial also showed a similar survival advantage for the nivolumab + ipilimumab combination [3]. Apart from these two combinations, the combinations of pembrolizumab + lenvatinib and nivolumab + cabozantinib have also demonstrated superiority over single-agent sunitinib and are recommended among first-line treatments [4,5].

The rapid integration of immunotherapies into treatment, particularly in combination with first-line therapy, has raised an important question: Which treatment should be selected for patients who progress on first-line therapy? Similarly, there is no standard approach for patients who progress on first-line VEGF-TKI therapy and receive second-line nivolumab. While reviews of patients who progress after ICI therapy suggest that re-treatment with ICI is not beneficial [6,7], various VEGF-TKIs have been used with limited success. No standard treatment recommendation has emerged [8,9,10,11]. In patients who have shown progression after immunotherapy, two VEGF-TKIs are particularly prominent: axitinib and cabozantinib. Axitinib is a tyrosine kinase inhibitor that inhibits VEGFR 1, 2, and 3 and has established its place in treatment guidelines with its efficacy as a single agent in patients with metastatic RCC who have progressed after one line of TKI [12]. Additionally, as mentioned above, it is also used in combination with pembrolizumab as a first-line treatment [2]. Cabozantinib, in addition to VEGFR, inhibits the mesenchymal–epithelial transition factor (MET), AXL, and rearranged during transfection (RET). Similar to axitinib, it has demonstrated efficacy in refractory disease, showing superiority over everolimus in the METEOR study. In the first-line setting, it has shown efficacy data both as a monotherapy in patients with intermediate to poor risk factors and in combination with nivolumab [5,13].

We conducted a study to present our real-world findings on patients treated with first-line VEGF-TKI, followed by second-line nivolumab. We aimed to determine the prognostic factors for progression and establish a treatment standard for patients progressing on this regimen. This is particularly important as there are often differences between patients selected for clinical trials and those encountered in real-world practice. Additionally, as is the case in our country, there is a need to inform treatment sequencing decisions in centers where first-line immunotherapy is unavailable.

## 2. Materials and Methods

### 2.1. Study Design and Patient Selection

Our study included patients with metastatic RCC who had received at least one line of TKI therapy, progressed on TKI treatment, and received second-line nivolumab therapy at Kartal City Hospital. Patients treated between January 2020 and January 2024 were included in our study. We recorded the patient characteristics, including age, ECOG performance status, metastatic sites, and IMDC scores. The IMDC prognostic risk group was computed based on the presence of six individual risk factors [i.e., <one year from the time of RCC diagnosis to first-line treatment initiation, Karnofsky Performance Scale < 80%, serum hemoglobin < lower limit normal (LLN), corrected calcium > upper limit normal (ULN), neutrophil count > ULN, platelet count > ULN] [14]. 

### 2.2. Endpoints

We also recorded which TKI therapies the patients had received before nivolumab and how many lines of TKI therapy they had undergone before switching to nivolumab treatment. The best response to nivolumab and the duration of the response were also noted. Toxicity related to our post-nivolumab treatment was assessed according to the Common Terminology Criteria for Adverse Events (CTCAE—Version 5.0) and the reasons for dose reduction or discontinuation of medication were recorded. The patients remained on therapy until disease progression or unacceptable toxicity. Baseline and subsequent radiographic assessments were performed, including CT or PET-CT scans, with brain and bone imaging as clinically indicated. Patient response was evaluated by our center’s nuclear medicine and radiology physicians according to RECIST 1.1 criteria. Standard blood work was checked once per 30-day cycle. The primary objective was to evaluate the efficacy of third-line TKI in patients with metastatic RCC progressing on prior nivolumab. The efficacy endpoints for the study were progression-free survival (PFS) and overall survival (OS).

### 2.3. Statistical Analysis

The data were analyzed statistically using SPSS 22.0 software (SPSS Inc., Chicago, IL, USA). Chi-square and Fisher’s exact tests were used for comparative data. The numerical variables between two independent conditions were analyzed using the Student’s t-test if they were normally distributed; otherwise, the Mann–Whitney U test was used. Progression-free survival (PFS) is defined as the time from the initiation of third-line systemic treatment until radiological progression, death, or the last visit date. Overall survival (OS) was defined as the time from the initiation of third-line systemic treatment until death from any cause or the last visit date. The overall and progression-free survival were calculated using Kaplan–Meier analysis. The effect of prognostic factors on survival was evaluated using the univariate log-rank test. The hazard ratio (HR) was calculated with a 95% confidence interval (CI). Multivariate analysis was performed using the Cox proportional hazards model to evaluate the effect of prognostic factors on survival. The significance level was set at ≤0.05.

### 2.4. Ethical Approval 

This study was performed in line with the principles of the Declaration of Helsinki. Approval was granted by the Kartal Dr. Lütfi Kırdar City Hospital, Istanbul, Türkiye, number 2024/010.99/2/18, 27 March 2024.

## 3. Results 

### 3.1. Baseline Characteristics

Our study investigated the efficacy and safety of third-line therapy following nivolumab in patients with metastatic RCC who progressed on prior nivolumab treatment. A total of 46 patients followed at our clinic received first-line VEGF-TKI therapy followed by second-line nivolumab. The baseline demographic characteristics are presented in Table 1. The median patient age was 59 years (range 19−87), and there were 38 (83%) males. Clear-cell RCC was the predominant histology (89%), and 27 (59%) patients had metastatic disease at diagnosis. Nephrectomy was performed in 33 patients (71%). IMDC risk stratification revealed 6 (13%) patients in the favorable group, 31 (67%) in the intermediate group, and 9 (20%) in the poor-risk group.

### 3.2. Efficacy

**Figure 1:**
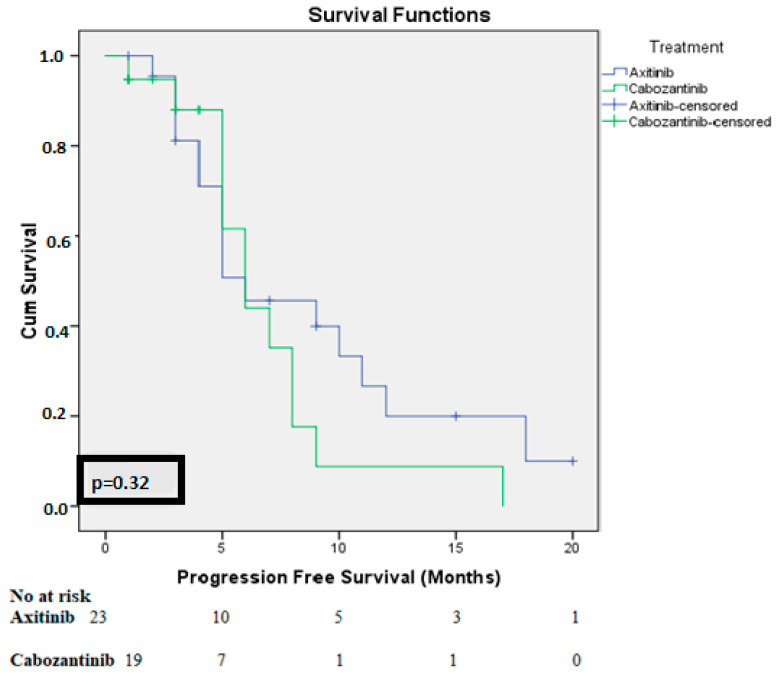
Kaplan–Meier curves describe similar progression-free survival differences between different treatment agents.

**Figure 2:**
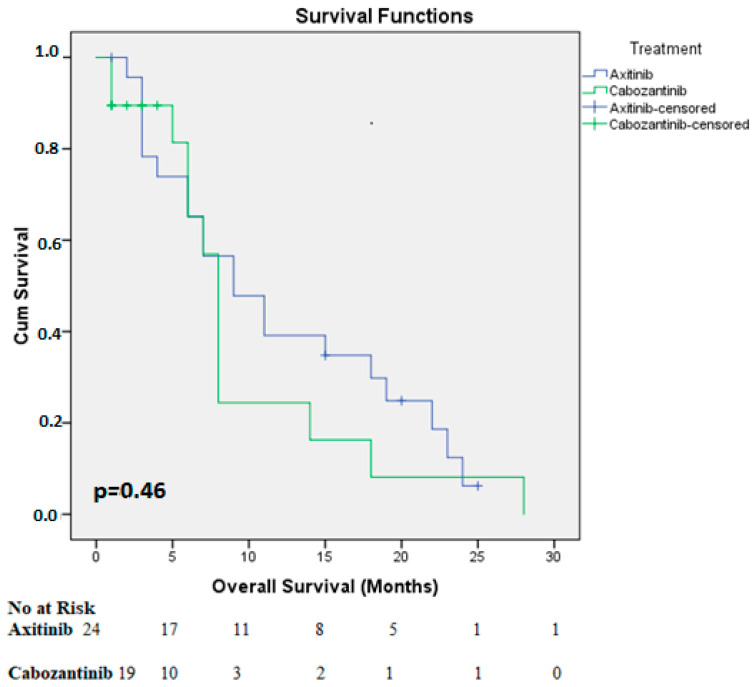
Kaplan–Meier Curves describe similar overall survival rates between different treatment agents.

Pazopanib (21 patients, 46%) and sunitinib (25 patients, 54%) were administered as a first-line therapy, with a median PFS of 10.2 months (range 2-80 months). All the patients received nivolumab in the second line, with a median of 12 cycles (range 5−61 cycles). The median second-line PFS was seven months (95% CI: 5.3−8.9 months). Third-line treatment regimens included axitinib (24 patients), cabozantinib (20 patients), and everolimus (2 patients). Details on the patient characteristics, efficacy, and survival data for this setting are provided in Table 2. Patients treated with everolimus were excluded from the survival analysis because there were only two such patients. The median PFS for patients receiving axitinib was 6 months, with 6-month and 12-month PFS rates of 45% and 23%, respectively. Cabozantinib demonstrated similar results (median PFS: 6 months; 6-month PFS: 43%; 12-month PFS: 8%). No statistically significant difference was observed between the two groups (*p* = 0.16). Notably, the two patients treated with everolimus had a poor prognosis, with a median PFS of only three months (Figure 1). The median overall survival from initiation of third-line therapy was eight months, with no statistically significant difference between the treatment arms (Figure 2). Cox regression analysis of patients who progressed on nivolumab and received third-line therapy (n = 46) identified the IMDC risk group and the ability to tolerate third-line therapy for at least six months as significant predictors of survival. De novo metastatic status, nephrectomy history, and first-line or third-line treatment agent selection did not significantly impact overall survival. However, it is important to acknowledge the limitations of the relatively small sample size.

### 3.3. Toxicity Evaluation

No unexpected, severe, or historically inconsistent drug-related toxicities were observed. The most common side effects associated with axitinib and cabozantinib were fatigue (28%) and hypertension (21%), followed by diarrhea (13%) and anemia (10%). Specific side effects for each agent included fatigue (40%) and diarrhea (30%) with cabozantinib and hypertension (29%) with axitinib. Notably, no treatment-related deaths were reported in the study (Table 3).

## 4. Discussion

The emergence of immunotherapy has significantly altered the treatment landscape for metastatic RCC, prompting a paradigm shift in treatment sequencing. Following the pivotal Checkmate-025 trial, which demonstrated improved survival with nivolumab in previously treated patients, pembrolizumab + axitinib and nivolumab + ipilimumab combinations have become standard first-line treatment options. However, identifying the optimal management strategy for patients who progress on immunotherapy remains a critical challenge.

Two prospective studies have addressed this challenge by exploring the efficacy of third-line treatment options. The CONTACT-03 trial evaluated the atezolizumab + cabozantinib combination in immunotherapy-pretreated patients, while a separate study investigated the efficacy of single-agent axitinib in this setting. The CONTACT-03 trial enrolled 522 patients and randomized them to atezolizumab + cabozantinib or single-agent cabozantinib [15]. While the study did not detect a statistically significant difference in efficacy between the arms, the combination arm exhibited a higher incidence of adverse events. This led to a recommendation against immunooncology-based (IO-based) therapy or IO-based combinations following IO-based treatment [16]. The second prospective study, a single-arm phase 2 trial, enrolled 40 patients and administered dose-titrated axitinib. Although the study did not meet its pre-specified statistical significance threshold, the authors reported promising activity in patients tolerating high doses.

Small retrospective studies evaluating the efficacy of TKIs following immunotherapy have reported treatment responses ranging from 16% to 41% [8,9,10,11]. Dudani et al. assessed the differential response rates between IO-IO and IO-TKI regimens, with IO-TKI demonstrating a lower response rate (14%) than IO-IO (45%) [17]. Subsequent analyses confirmed this difference [18]; however, it is important to note that nearly half of the IO-TKI patients in this update analysis received sunitinib or pazopanib. Data on the efficacy of these agents beyond the first-line setting are limited.

Three retrospective studies, including our own, have investigated single-agent TKI or everolimus treatment in patients progressing after single-agent immunotherapy [9,19,20]. Graham et al. reviewed six TKIs and two mTOR inhibitor studies in post-immunotherapy settings [15]. The most frequently used agents, axitinib, cabozantinib, and sunitinib, demonstrated median treatment-to-discontinuation times (TTD) of 10.2, 11.4, and 5.5 months, respectively, and one-year overall survival (OS) rates of 89%, 83%, and 78%, respectively. Notably, while the efficacy of cabozantinib and sunitinib remained relatively stable across treatment lines, axitinib exhibited a significant decline in efficacy in later lines. Additionally, the one-year OS was significantly lower in the TKI arms compared to the mTOR arm. Powles et al. compared cabozantinib and everolimus in the METEOR trial, demonstrating a clear superiority of cabozantinib in this setting [20]. Albiges et al. presented data on five TKIs and two mTOR inhibitors used following immunotherapy progression, reporting a significant advantage for TKI over mTOR therapies [9]. Axitinib emerged as the most effective agent, with a one-year survival rate of 69% compared to 27% for everolimus.

Our study deviated from the literature regarding overall survival and progression-free survival outcomes, which were shorter than those reported in the aforementioned retrospective studies. This discrepancy may be due to our patients having received third-line therapy, and all of them had previously received at least one line of TKI treatment. While the number of patients receiving cabozantinib and axitinib was balanced in our data, the limited number of patients treated with everolimus precludes definitive conclusions regarding this agent. However, the use of everolimus, particularly in the context of newer-generation TKIs and immunotherapies, has declined in real-world practice.

Most studies investigating VEGF-TKIs following immunotherapy have not reported novel safety concerns, with toxicity profiles resembling those observed in the first-line setting. However, pazopanib has been associated with severe liver toxicities when used in combination with immunotherapy or following immunotherapy progression [10]. In heavily pre-treated patients, particularly those with intermediate or poor prognoses, grade 3/4 fatigue, hand-foot syndrome, diarrhea, and hypertension are more prevalent. In our series, we did not observe any agent-specific adverse events. Hypertension was the primary side effect associated with axitinib, while fatigue and diarrhea were most common with cabozantinib. However, these were manageable without significant complications.

## 5. Conclusions

To our knowledge, there is no standard approach after progression in patients receiving nivolumab in the second series after the first series of TKI in the treatment of metastatic RCC, and the data in the literature are quite limited. In the limited prospective studies conducted after immunotherapy, robust data to establish a standard with VEGF-TKI or IO-based treatments have not been obtained. However, multiple retrospective studies have reported minimal efficacy with axitinib and cabozantinib. In our study, we demonstrated with our data that axitinib and cabozantinib are two treatment agents that can be recommended at this stage. Both treatment agents exhibited a 6-month progression-free survival. Additionally, both treatment agents have manageable toxicity profiles. In our study, known side effects of the drugs were observed, and no new adverse effects were detected. Our efficacy and toxicity data are consistent with the literature. However, the fact that our study is retrospective and includes a small number of patients stands out as our limitations, and strong prospective data are needed to provide standard recommendations in this field. 

## Figures and Tables

**Table 1:** Baseline demographic findings.

Variables		n = 46 (%)
Age (median) (years)		59 (range 19−87)
Gender	Female	8 (17)
	Male	38 (83)
Pathologic subtype	Clear cell	41 (89)
	Papillary	4 (9)
	Sarcomatoid	1 (2)
Stage at diagnosis	Stage 1	3 (6)
	Stage 2	5 (11)
	Stage 3	11 (24)
	Stage 4	27 (59)
Nephrectomy	Present	33 (71))
	Absent	13 (29)
IMDC * Risk score	0	6 (13)
	1	19 (41)
	2	12 (26)
	3	6 (13)
	4	3 (7)
IMDC * Risk group	Favorable	6 (13)
	Intermediate	31 (67)
	Poor	9 (20)
First-Line treatment	Pazopanib	21 (46)
	Sunitinib	25 (54)
First-line progression-free survival (median months)		10.2 (range 2−80)
Nivolumab dosing (median cycles)		12 (range 5−61)
Progression-free survival for nivolumab (median months)		7 (95%CI: 5.3−8.9 months)

* IMDC prognostic risk groups were calculated by adding prognostic risk factors that included the following: 1. < 1 year from time of diagnosis to first-line therapy; 2. Karnofsky performance status < 80%; 3. Hemoglobin < LLN; 4. Calcium > ULN; 5. Neutrophil > ULN; 6. Platelets > ULN. Patients with 0 risk factors were categorized as favorable, 1–2 risk factors as intermediate, and 3 or more risk factors as poor.

**Table 2:** Post-nivolumab treatment characteristics and survival outcomes.

Variables, (n = 46)					n (%)			
Treatment				Axitinib	24 (52)			
				Cabozantinib	20 (43)			
				Everolimus	2 (5)			
Progression (n = 46)				Absent	15 (33)			
				Present	31 (67)			
Progression at treatment groups	Axitinib			Absent	7 (29)		*p* = 0.47	
				Present	17 (71)			
	Cabozantinib			Absent	8 (40)			
				Present	12 (60)			
	Everolimus			Absent	0			
				Present	2 (100)			
PFS * (months)				Median (95%CI)	6 (5.0−6.99)			
				6 months rate (%)	42			
				12 months rate (%)	17			
PFS * at treatment groups (months)		Axitinib		Median (95%CI)	6 (2.4−9.5)	*p* = 0.32		
				6 months rate (%)	45			
				12 months rate (%)	23			
		Cabozantinib		Median (95%CI)	6 (4.3−7.6)			
				6 months rate (%)	43			
				12 months rate (%)	8			
Post-progression treatment				Best supportive care	16 (53)			
				Axitinib	3 (10)			
				Cabozantinib	5 (16)			
				Everolimus	6 (21)			
Status				Alive	12 (26)			
				Exitus	34 (74)			
OS ^†^ (months)				Median (95%CI)	8 (6.7−9.3)			
				12 months rate (%)	35			
				24 months rate (%)	8			
OS ^†^ at treatment groups (months)			Axitinib	Median (95%CI)	9 (4.3−13.6)			*p* = 0.46
				12 months rate (%)	52			
				24 months rate (%)	6			
			Cabozantinib	Median (95%CI)	8 (7.2−8.7)			
				12 months rate (%)	24			
				24 months rate (%)	8			

* PFS: progression-free survival, † OS: overall survival. Overall and progression-free survival were calculated using Kaplan–Meier analysis. A *p*-value < 0.05 was considered statistically significant.

**Table 3:** Adverse events.

	All Patients n (%)	Treatment n (%)		
		Cabozantinib	Axitinib	*p*
Fatigue	13 (28)	8 (40)	4 (16)	0.08
Anemia	4 (10)	4 (20)	1 (4)	0.02
Hand and foot syndrome	3 (6)	3 (15)	1 (4)	0.21
Hypertension	10 (21)	3 (15)	7 (29)	0.26
Diarrhea	6 (13)	6 (30)	0	0.04
Other	8 (17)	6 (30)	2 (8)	0.06

## Data Availability

The raw data supporting the conclusions of this article will be made available by the authors upon request.
